# Multivariate analysis of the effect of Chalazia on astigmatism in children

**DOI:** 10.1186/s12886-022-02529-1

**Published:** 2022-07-17

**Authors:** Lijuan Ouyang, Xinke Chen, Lianhong Pi, Ning Ke

**Affiliations:** Department of Ophthalmology, Children’s Hospital of Chongqing Medical University, National Clinical Research Center for Child Health and Disorders, Ministry of Education Key Laboratory of Child Development and Disorders. China International Science and Technology Cooperation base of Child Development and Critical Disorders. Chongqing Key Laboratory of Pediatrics, 136 zhongshan 2nd RD, Yuzhong District, Chongqing, 400014 China

**Keywords:** Astigmatism, Amblyopia, Chalazion, Double-angle plots, Preschool children

## Abstract

**Background:**

Chalazion may affect visual acuity. This study aimed to evaluate refractive status of chalazia and effect of different sites, sizes, and numbers of chalazion on astigmatism.

**Methods:**

Three hundred ninety-eight patients aged 0.5–6 years were divided into the chalazion group (491 eyes) and the control group (305 eyes). Chalazia were classified according to the site, size, and number. Refractive status was analyzed through the comparison of incidence, type, mean value and vector analysis.

**Results:**

The incidence, type, refractive mean and of astigmatism in the chalazion group were higher than those in the control group, and the difference was statistically significant (*P* < 0.05). For comparison of the incidence, the middle-upper eyelid (50%) was highest, followed by 41.77% in the medial-upper eyelid, both higher than that in the control group (*P* < 0.05). In medium (54.55%) and large groups (54.76%) were higher than that in the control group (27.21%) (*P* < 0.05). In multiple chalazia, the astigmatism incidence for chalazion with two masses was highest (56%), much higher than that in the control group (*P* < 0.05). However, this difference was not significant in chalazion with ≥3 masses (*P* > 0.05). For comparison of the refractive mean,the medial-upper eyelid, middle-upper eyelid and medial-lower eyelid were higher than the control group (*P* < 0.05) (*P* < 0.05). The 3-5 mm and >5 mm group were higher than those in the control group and <3 mm group(*P* < 0.05), and the>5 mm group was larger than the 3-5 mm group，suggesting that the risk of astigmatism was higher when the size of masses > 5 mm. Astigmatism vector analysis can intuitively show the differences between groups, the results are the same as refractive astigmatism.

**Conclusion:**

Chalazia in children can easily lead to astigmatism, especially AR and OBL. Chalazia in the middle-upper eyelid, size ≥3 mm, and multiple chalazia (especially two masses) are risk factors of astigmatism. Invasive treatment should be performed promptly if conservative treatment cannot avoid further harm to the visual acuity due to astigmatism.

**Supplementary Information:**

The online version contains supplementary material available at 10.1186/s12886-022-02529-1.

## Background

A chalazion is an idiopathic chronic nonpurulent inflammation of the meibomian gland that progresses slowly with pressing pain. A deep chalazion is caused by inflammation of the meibomian gland, whereas a superficial chalazion is caused by inflammation of the Zeis gland [1]. A chalazion causes swelling of the eyelids and ptosis and can develop in children and adults. Several risk factors have been reported, including local factors such as eyelid mite infection [2], meibomian gland dysfunction and blepharitis [3, 4], and rosacea in children [5], as well as systemic factors such as vitamin A deficiency [6], low serum ferritin levels [7], and androgen imbalance in adolescents [8]. Currently, chalazion masses have various treatment options. Conservative treatment includes hot compresses and/or combined with medications such as tobramycin or tobramycin dexamethasone eye drops and ophthalmic ointment [9]. If conservative treatment fails, invasive treatment such as local steroid injections or surgical excision can be performed [10].

Some common eye diseases, such as congenital ptosis [11], eyelid hemangioma [12], allergic conjunctivitis [13] and bad eye rubbing habit [14], can cause astigmatism in children. It has been confirmed that large chalazions in the middle- upper eyelid could cause corneal astigmatism [15–18]. In an elderly man with farsightedness due to a chalazion mass in the middle-upper right eyelid, visual acuity returned to normal after the mass was removed [19]. In another case, a patient undergoing laser-assisted in situ keratomileusis had an upper eyelid chalazion that resulted in the loss of visual acuity [20].

Astigmatism is a common type of refractive error, and its effect on visual acuity is determined based on both dioptric and axial aspects [21]. Traditional methods compare astigmatism mainly according to the incidence and degree of astigmatism, ignoring the influence of astigmatism direction. Eydelman MB et al. introduced vector analysis to calculate eye astigmatism changes [22]. For astigmatism screening in younger children, the American Academy of Pediatrics Committee on Ophthalmology and Strabismus (AAPOS) recommends using the Spot binocular visual acuity screener that can screen children aged over 6 months and has a high specificity for children with refractive abnormalities (including astigmatism) and high-risk factors for amblyopia [23, 24].

Most studies on the relationship between chalazion and astigmatism have focused on corneal astigmatism rather than total astigmatism [15, 17, 18]. However, these studies have reported single-site masses, mainly larger masses in the middle-upper eyelid, and lack comprehensive studies of all site [15, 16]. Moreover, the aforementioned studies have included mainly older children (> 7 years old) [25] or adults [15–17, 19] with no reports on preschool children (except for case reports), and the sample size is small [15, 16, 18]. However, children aged 1–6 years old are more likely to develop chalazion, especially those between 2 and 5 years old [3]. Nevertheless, no large-sample studies have examined the effects of chalazion masses on the incidence rate and type of astigmatism in children. To the best of our knowledge, this is the first study to investigate the features of astigmatism in a large sample of children aged 0–6 years with chalazion masses. By the comparison of incidence, type, mean value and vector analysis of astigmatism to investigate the effect of the site, size, and the number of chalazion masses in astigmatism and to clarify the risk factors of chalazion-induced astigmatism.

## Material and methods

### Participants

A total of 398 children aged 0.5–6 years who complained of chalazion masses and attended the Department of Ophthalmology of the Children’s Hospital of Chongqing Medical University from June 2020 to June 2021 were included in the study. All children were distributed into the chalazion group (491 eyes) and the non-chalazion group (control group, 305 eyes) according to their chalazion status. The sites, sizes, and numbers of chalazion masses in each eye were recorded and classified, and the refractive status was examined. Chalazion masses were also divided into the single chalazion group and the multiple chalazion group (≥2) according to the number of chalazion masses. Moreover, single chalazion masses were classified into the following six groups according to the location in the eyelid: lateral-upper, middle-upper, medial-upper, lateral-lower, middle-lower, and medial-lower eyelids. Finally, single chalazion cases were divided into three groups according to the size of the masses: small (< 3 mm), medium (3–5 mm), and large (> 5 mm) [15]. Patients who met the following inclusion criteria were included: (1) consent of the child’s parents or legal guardians for participation in the study, good compliance of the child, and signed informed consent form; (2) children aged 0–6 years diagnosed with chalazion masses in one or both eyes; and (3) no allergic conjunctivitis, trichiasis or often rubbing the eye and other symptoms; (4) no opacity of the refracting media and no other ophthalmic diseases such as strabismus. The exclusion criteria were as follows: (1) children aged ≥6 years; (2) eyelid abnormalities such as congenital or acquired ptosis, lower eyelid entropion and trichiasis, and eyelid masses other than chalazion; (3) corneal abnormalities such as ocular trauma, history of wearing contact lens, keratoconjunctival dermoid tumor, corneal scarring, and severe xerophthalmia; and (4) children with a history of diseases other than a chalazion and who were uncooperative in the examination of visual acuity. The study protocol was approved by the ethics committee of the Children’s Hospital of Chongqing Medical University. The protocol adhered to the provisions of the Declaration of Helsinki.

### Research methods

All children underwent a routine ophthalmic examination by the same physician, who palpated for masses and then everted the lids to check if there were any chalazion on the conjunctival surface of the eyelid and recorded the number, size, and sites of chalazion masses in each eye. They also underwent an astigmatic diopter examination.

### Measurement of the size of chalazion masses

**Fig. 1:**
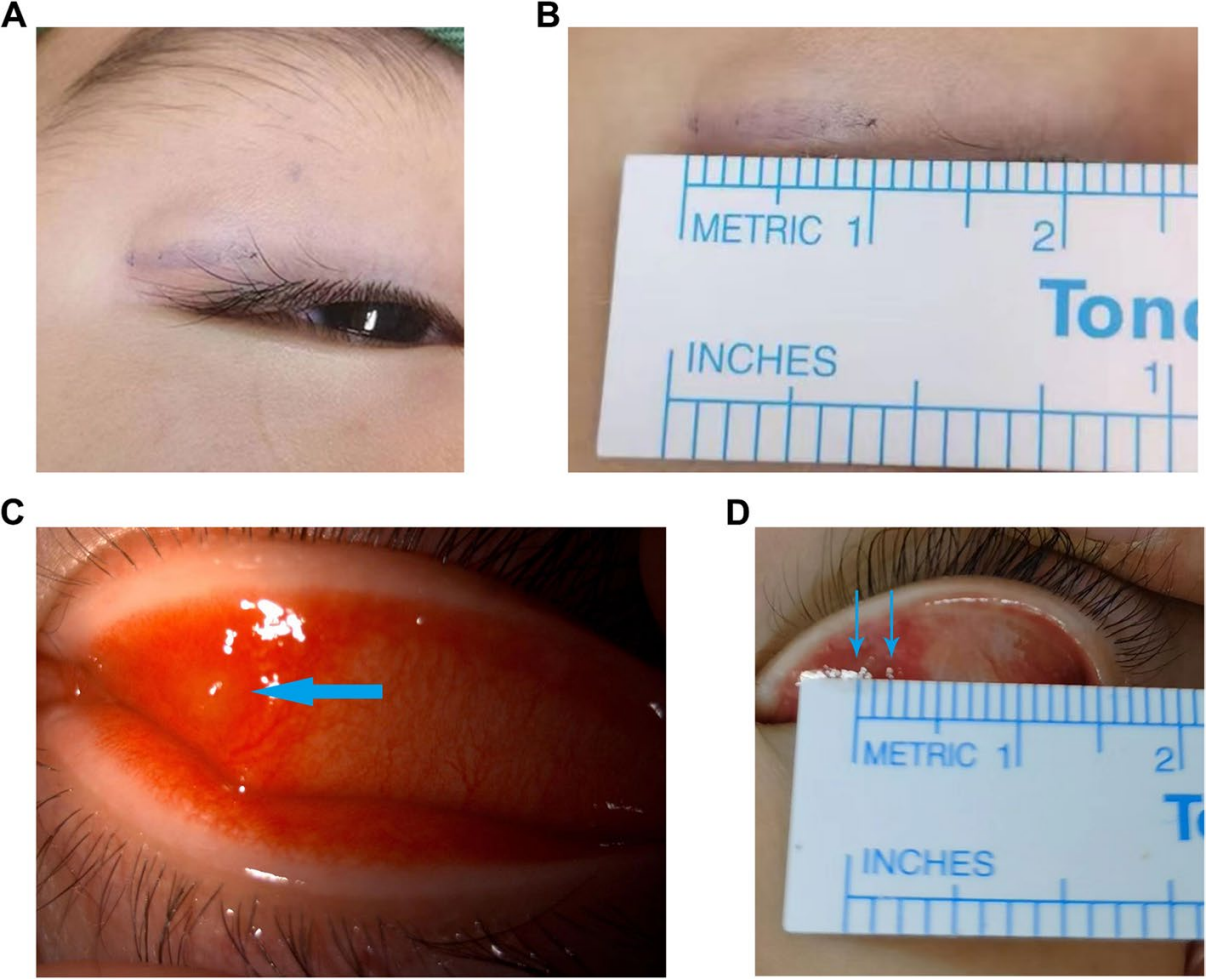
Measurement of the size of chalazion masses. **A** Chalazion protruding from the skin surface, marked the edge of the mass with gentian violet. **B** Measured it with a ruler (the horizontal diameters = 9 mm). **C** A chalazion with no obvious tactile sensation, we everted the eyelid to evaluated the edge of the mass according to the morphology and color changes of the conjunctival surface. **D** Measured it with a ruler (the horizontal diameters = 2 mm)

For the chalazia masses protruding from the skin surface, we marked the edge of the masses with gentian violet on the skin surface (Fig. 1A), for the chalazia masses with no obvious tactile sensation, we evaluated the edge of the lump according to the morphology and color changes of the conjunctival surface of the eyelid after opening the eyelid (Fig. 1C) and then measured the horizontal and vertical diameters of the mass with a ruler (Fig.1B,D) [18]. Given the three-dimensional structure of chalazion masses, the total volume of each mass is theoretically the most clinically significant measurement. However, considering the difficulty of three-dimensional measurement, we used the horizontal width of each mass as a proxy for this value [9].

### Astigmatic diopter and axial measurement

Astigmatic diopter and axial parameters were measured three times by a Spot binocular visual acuity screener (Spot Vision Screener Model VS100, Welch Allyn, Skaneateles Falls, NY, USA) [23, 24] and averaged.

### Data collection and processing

To reduce error, data collection and processing were performed by the same physician, and astigmatism was defined by an absolute value of ≥1.00 degrees in cylindrical power. Data were analyzed on an eye-by-eye basis. WR was defined as astigmatism with the main meridian of maximum refractive power at 90° ± 30°, AR at 180° ± 30°, and OBL between 30° and 60° or 120° and 150°. In this study, non-WR refers to the sum of AR and OBL.

### Vector analysis and doubled-angle plots

Convert all manifest refraction data from the spectacle to the corneal plane. Flip the cylinder axes of left eyes around the vertical axis so that errors due to cyclotorsion or antisymmetrical healing patterns do not tend to cancel out when averaging data from right and left eyes. The correct conversion method is to create a “transformed” refraction for left eyes in which the new axis is equal to 180° minus the original axis [22]. The usual plotting convention is to label the polar plot with axes from 0° to 180° (based on axes prior to doubling) instead of 0° to 360°. A cylinder vector C is defined as C = (x, y), with its absolute C = |C|. A = axis(°). The vector components are calculated as follows: x = C × cos(2 × A), y = C × sin(2 × A) (Holladay). The centroid values of each plotted vector in the ophthalmic coordinate system X_CENTROID_= $\frac{\sum_{i=1}^n Xi}{n}$ and Y_CENTROID_= $\frac{\sum_{i=1}^n Yi}{n}$ (n = the number of eyes). The length of ellipse in horizontal semi-axis = standard deviation of X= $\sqrt{\frac{\sum_{i=1}^n\left( Xi-X\right)}{n-1}}$,length of ellipse in vertical semi-axis = standard deviation of Y= $\sqrt{\frac{\sum_{i=1}^n\left( Yi-Y\right)}{n-1}}$ [22, 26]. The doubled-angle plot is a polar plot of astigmatism data using the value of the cylinder for the magnitude and the axis of the astigmatism for the angle. The angles range from 0 to 180 degrees and correspond to the range of angles for astigmatism. The rings represent the magnitude of the astigmatism; the inner ring is 1.0 D and the step size between rings 1.0 D [27].

### Statistical analysis

All data were categorized, recorded, and tabulated. SPSS software version 20.0 (IBM Corp., Armonk, NY, USA) was used for all statistical analyses. The χ2 test was used to compare the incidence rates of astigmatism types, and the Fisher exact probability method was used when the theoretical frequency number of row x list exceeded 20% < 5. Student’s t-test and one-way ANOVA were applied in the comparison of differences between two or three groups of measurement data, respectively. The data were presented as mean ± SD. The difference was considered significant when *P* < 0.05. When multiple groups showed a significant difference, further two-by-two comparisons were performed three times at a test level of α = 0.05/3 = 0.0167.

## Results

### General information

A total of 796 eyes of 398 children (male, *n* = 167; female, *n* = 231) were included in the study. The youngest and oldest participants were 6 months and 71 months, respectively (mean age, 2.86 ± 1.301 years). Of the total eyes, 491 had a chalazion, while 305 had no chalazion. There were 345 eyes (70.26%) with single chalazion and 146 eyes (29.74%) with multiple chalazion masses. For solitary chalazion, 1) according to site of the chalazion, differences in the distribution were noted in the lateral-upper (*n* = 64), middle-upper (*n* = 98), medial-upper (*n* = 79), lateral-lower (*n* = 38), middle-lower (*n* = 41), and medial-lower (*n* = 25) eyelids. More chalazion masses were observed in the upper eyelid than in the lower eyelid and in the middle eyelid than in the lateral and medial eyelids. Meanwhile, the highest number of chalazion masses was in the middle-upper eyelid. 2) according to size of the chalazion, small group (*n* = 159), medium group (*n* = 143) and large group (*n* = 43). In multiple chalazia: 1) according to site of the chalazion, the upper eyelid (*n* = 59), the lower eyelid (*n* = 53), and both eyelids (*n* = 112), 2) according to number of the chalazion, two masses (*n* = 100), more than three masses (*n* = 46) (Table 1).

**Table 1:** Baseline distribution of the 798 eyes

	Cases		Cases		Cases
Control	305				
Chalazion group	491				
Site (single)		Size (single)		Number	
Lateral-upper	64	Small (< 3 mm)	159	Single	345
Middle-upper	98	Medium (3–5 mm)	143	Muitiple	146
Medial-upper	79	Large (> 5 mm)	43	2	100
Lateral-lower	38			≥3	46
Middle-lower	41				
Medial-lower	25				

### Incidence, types of astigmatism between the two groups and different groupings

The incidence rate of astigmatism in the chalazion group was 42.97% (211/491), which was significantly higher than that in the control group (27.21%, 83/305) (χ2 = 20.062, P < 0.001) (Table 2). In both groups, WR was the predominant type of astigmatism. The incidence rates of the different types of astigmatism in the two groups were 72.98% versus 83.34% for WR, 19.43% versus 13.25% for AR, and 7.58% versus 2.41% for OBL. The incidence rate of OBL in the chalazion group was 27.02%, which was significantly higher than that in the control group (16.66%) (χ2 = 4.231, *P* = 0.04) (Table 3).

**Table 2:** Comparison of the incidence rate of astigmatism between the two groups

Chalazion	Total	Astigmatism		χ^2^	P
		Yes	No		
Yes	491	211	280	20.062	0.000*
No	305	83	222		
Total	796	294	502		

* indicates a difference in the incidence rate of astigmatism between the chalazion group and the control group (*P* < 0.05)

**Table 3:** Comparison of the incidence rates of different astigmatism types between the two groups

Chalazion	Total	Types		χ^2^	*P*
		WR	Non-WR (AR + OBL)		
Yes	211/491	154	57 (41 + 16)	4.231	0.04*
No	83/305	70	13 (11 + 2)		
Total	241/796	224	70 (52 + 18)		

* indicates a difference in the incidence rate of different types of astigmatism in the chalazion and control group (*P* < 0.05). WR = with-the-rule astigmatism; AR = against-the-rule astigmatism；OBL = oblique astigmatism; non-WR = AR + OBL

**Fig. 2:**
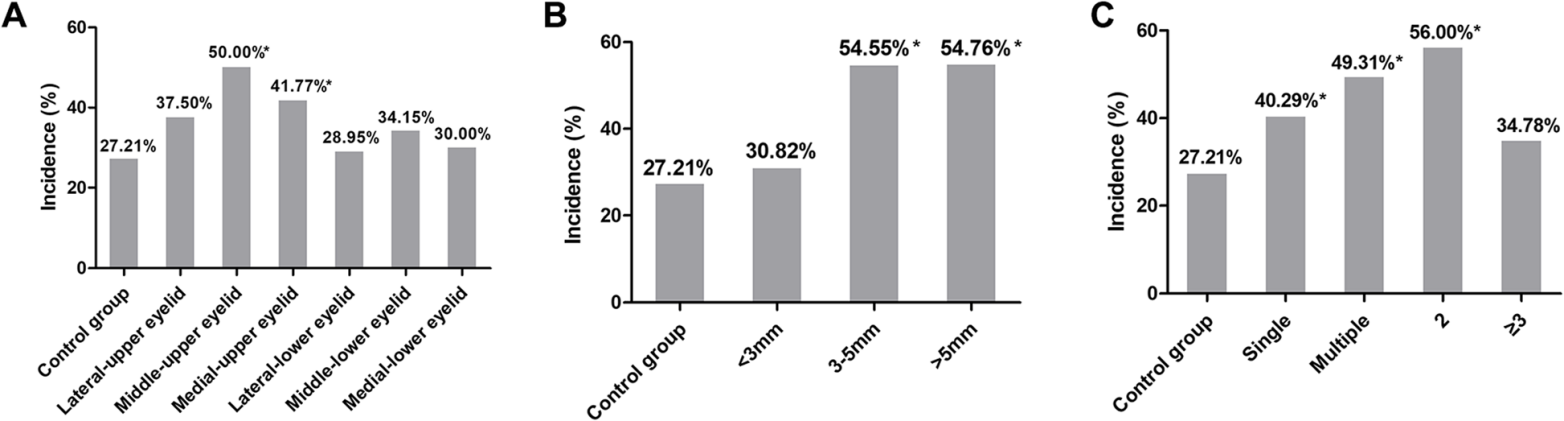
Incidence of astigmatism according to different groupings. **A** Comparison of the incidence rate of astigmatism in patients with a single chalazion at different sites. **B** Comparison of the incidence rate of astigmatism by mass size in patients with a single chalazion. **C** Comparison of the incidence rate of astigmatism between groups with specific numbers of chalazion masses. *indicates a difference in the incidence rate of astigmatism (*P* < 0.05)

According to the site, in a solitary chalazion, the highest incidence rate of astigmatism was 50% (49/98) in the middle-upper eyelid group, 41.77% (33/79) in the medial-upper lid group, 37.50% in the lateral-upper eyelid group, 34.15% in the middle-lower lid group, 30.00% in the medial-lower eyelid group, and 28.95% in the lateral-lower eyelids, with statistically significant differences (χ^2^ = 20.343 *P* = 0.002). The difference between the incidence rates of astigmatism in the medial-upper and middle-upper eyelid groups with that of the control group were statistically significant (*P* < 0.001 and *P* = 0.01, respectively). In conclusion, the incidence rate of astigmatism was higher in the upper eyelid group than in the lower eyelid group, higher in the medial eyelid group than in the lateral eyelid group, and highest in the middle eyelid group (Fig. 2A). According to the size, The incidence rate of astigmatism was significantly higher in the 3–5 mm (54.55%) and the > 5 mm group (54.76%) than that in the < 3 mm group (30.82%) and the control group (27.12%) (χ2 = − 5.661 *P* = 0 .000). Moreover, we found a statistically significant difference in the incidence rate of astigmatism between the 3–5 mm and > 5 mm groups with that of the control group (*P* < 0.001). Further, the incidence rate of astigmatism was higher than 50% when the mass size was ≥3 mm (Fig. 2B). According to the number，When the number of masses was 2, the highest incidence rate of astigmatism was 56%. Further, in this case, the astigmatism incidence rate was significantly higher in the multiple chalazion group (49.31%) than that in the solitary chalazion group (40.29%) both higher than that in the control group (27.21%; χ2 = 20.343, *P* = 0.002). However, pairwise comparisons showed that the difference in the incidence rate of astigmatism was not statistically significant when the number of masses was ≥3 (*P* > 0.05) (Fig. 2C).

### Refractive astigmatism between the two groups and different groupings

**Fig. 3:**
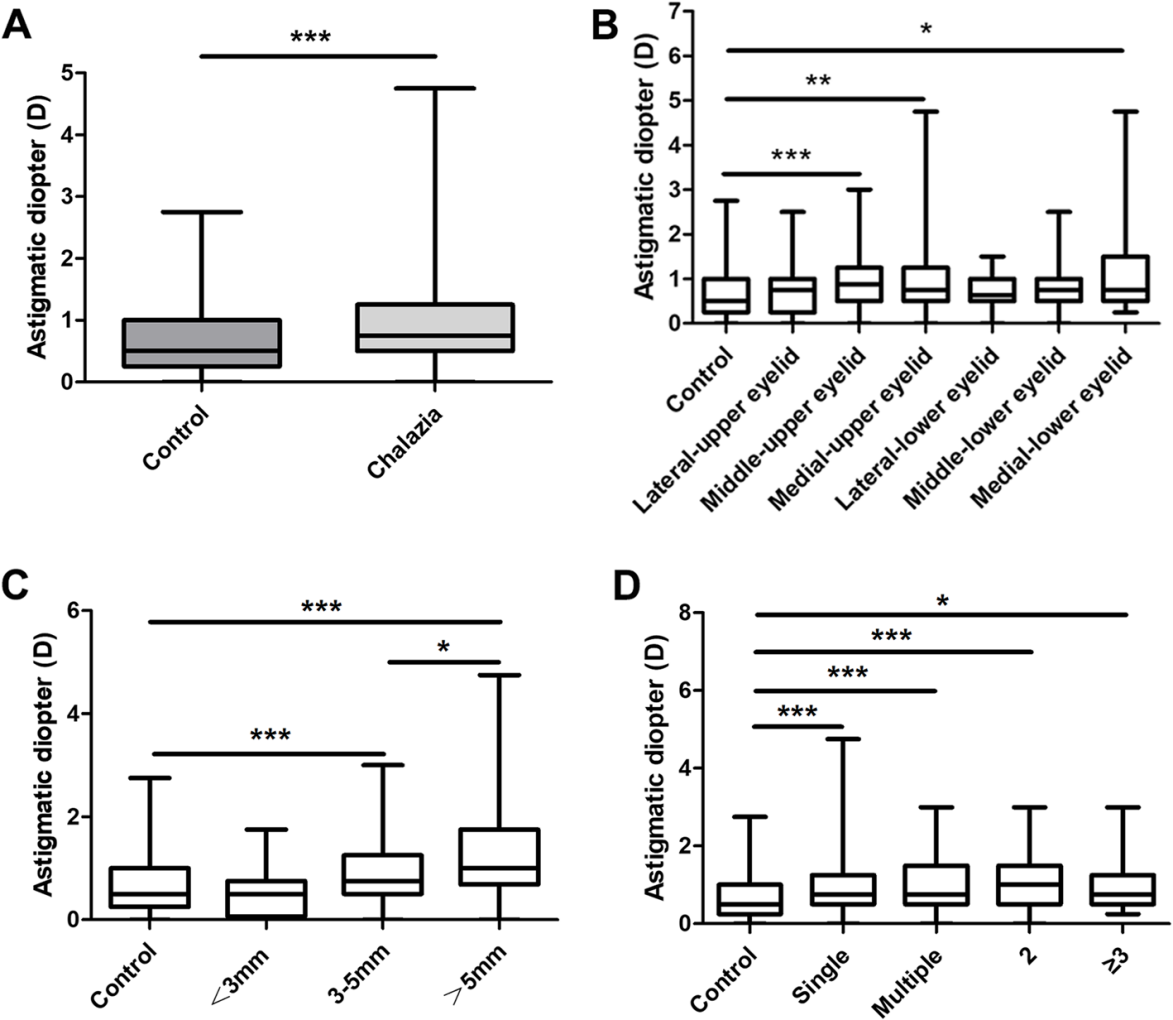
Box-whisker plots of refractive astigmatim according to different groupings. **A** Box-whisker plots of refractive astigmatim between control group and chalizia group. **B** Box-whisker plots of refractive astigmatim in patients with a single chalazion at different sites. **C** Box-whisker plots of refractive astigmatim by mass size in patients with a single chalazion. **D** Box-whisker plots of refractive astigmatim between groups with specific numbers of chalazion masses. *indicates a difference in the incidence rate of astigmatism (*P* < 0.05)

Refractive astigmatism between the two groups and different groupings are presented in Fig. 3 and Table 4. The arithmetic mean of astigmatism was greater in the chalazion group compared with the control group (*p* < 0.001). According to different sites in a solitary chalizion, the mean value of middle-upper, medial-upper, medial-lower was greater compared with the control group (*p* = 0.001, *p* = 0.005, *p* = 0.02, respectively). According to different sizes in a solitary chalizion, the mean value in medium and large group was significantly greater than that in the small and control group (*p* = 0.001, *p* = 0.000, respectively). Moreover, the mean value in large group was greater in medium group (*p* = 0.032). According to numbers, the mean value in all goups were greater than that in the control group (*p*<0.05).

**Table 4:** Comparison of astigmatism of chalazion

	Astigmatic diopter (D)	*P*
Control group	0.68 ± 0.53	< 0.0001
Chalazion group^a^	0.90 ± 0.65	
Single chalazion according to different locations		
Control group	0.68 ± 0.53	
Lateral-upper eyelid	0.79 ± 0.64	
Middle-upper eyelid^a^	0.95 ± 0.59	0.001
Medial-upper eyelid^a^	0.93 ± 0.76	0.005
Lateral-lower eyelid	0.64 ± 0.41	
Middle-lower eyelid	0.74 ± 0.52	
Medial-lower eyelid^a^	1.04 ± 0.95	0.022
Single chalazion according to different sizes		
Control group	0.68 ± 0.53	
Small (< 3 mm)	0.53 ± 0.46	
Medium (3–5 mm)^a^	0.90 ± 0.61	0.001
Large (> 5 mm)^ab^	1.19 ± 0.88	0.000, 0.032
According to numbers		
Control group	0.68 ± 0.53	
Single	0.86 ± 0.66	
Muitiple^a^	1.00 ± 0.62	0.000
2^a^	1.01 ± 0.61	0.000
≥ 3^a^	0.96 ± 0.65	0.015

^a^means a difference in the mean astigmatic diopter compared with the control group (*P* < 0.05). ^b^means a difference in the Mean astigmatic diopter compared with the medium (3–5 mm) group (*P* < 0.05)

### Astigmatism vector analysis and double-angle plots

**Fig. 4:**
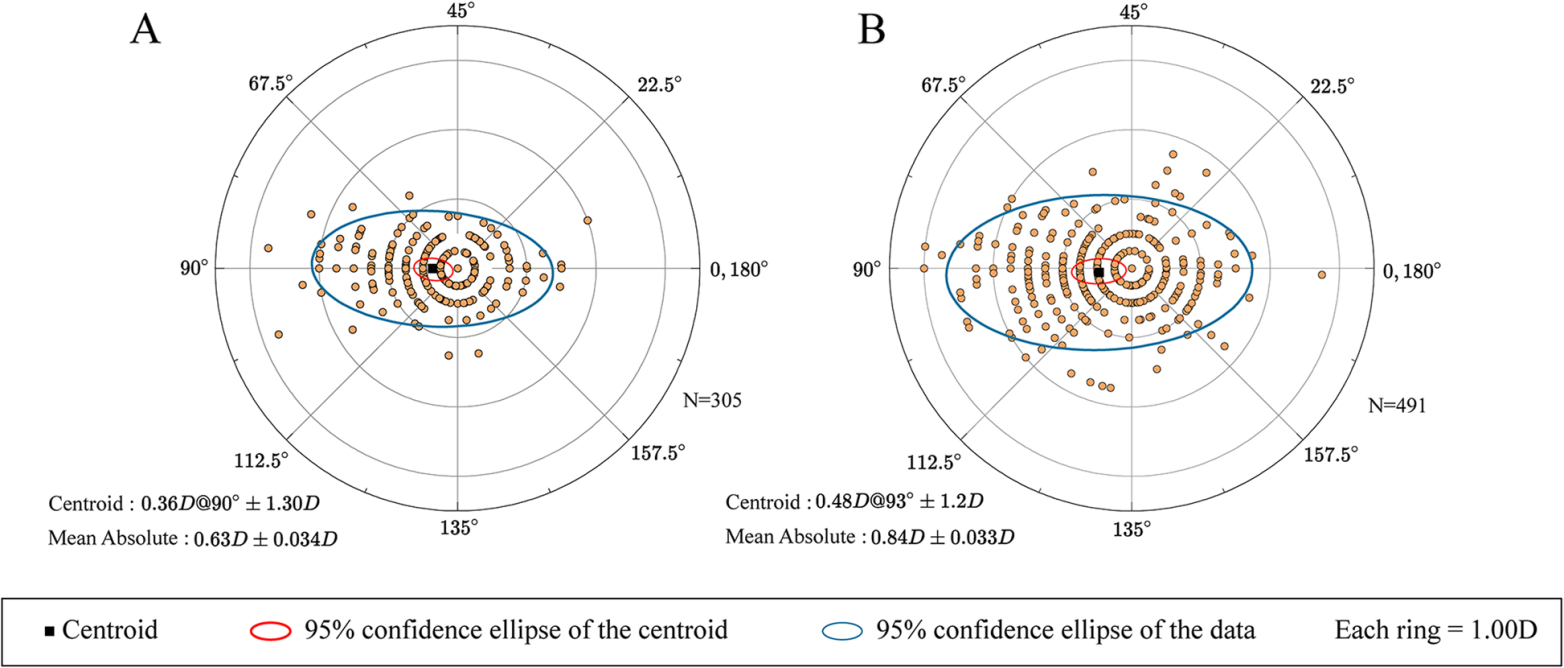
A distribution of vector astigmatism in a Double-angle plots between chalazion and control group. **A** = control group, **B** = chalazion group

**Fig. 5:**
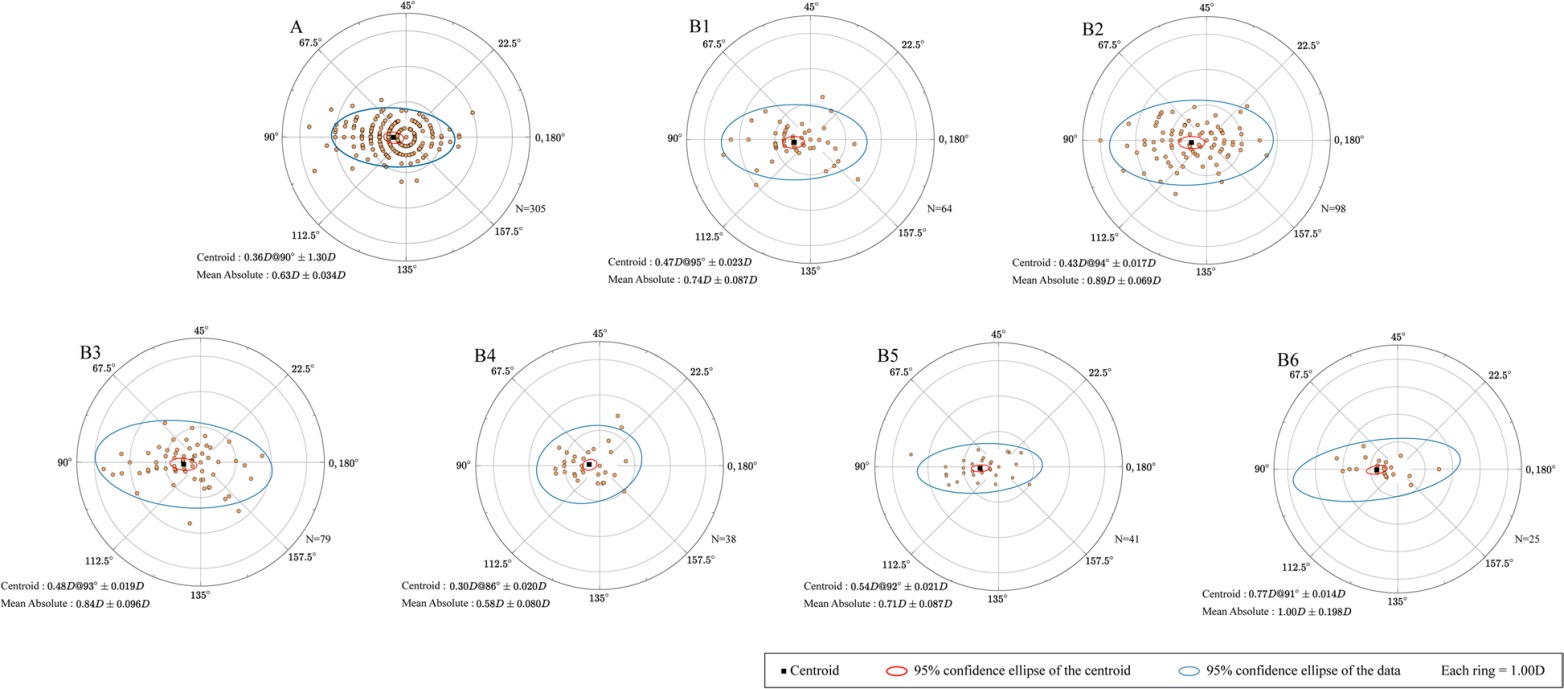
A distribution of vector astigmatism in a Double-angle plots in patients with a single chalazion at different sites. A = control group, B1 = Lateral-upper eyelid, B2 = Middle-upper eyelid, B3 = Medial-upper eyelid, B4 = Lateral-lower eyelid, B5 = Middle-lower eyelid, B6 = Medial-lower eyelid

**Fig. 6:**
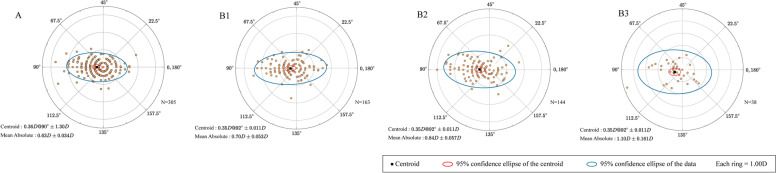
A distribution of vector astigmatism in a Double-angle plots by mass size in patients with a single chalazion. A = control group, B1 = <3 mm group, B2 = 3-5 mm group, B3 = >5 mm group

**Fig.7:**
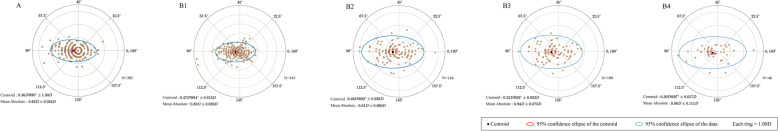
A distribution of vector astigmatism in a Double-angle plots between groups with specific numbers of chalazion masses. A = control group, B1 = single group，B2 = Muitiple group, B3 = masses (2) group，B4 = masses (≥3) group

The results of vector analysis of astigmatism are presented in Figs. 4, 5, 6, 7. Double angle plots can intuitively display the mean value and centroid value of astigmatism vector, 95% confidence ellipse of the centroid. Figure 4 shows the double angle plots between the chalazion and the control group, we can conclude that chalazion is more likely to cause astigmatism and the degree is more serious. Figure 5 shows that chalazion in the upper eyelid has a great influence on astigmatism. Figure 6 shows that chalazion in the medium and large group have a great influence on astigmatism. Figure 7 shows that multiple chalazia has a great influence on astigmatism.

## Discussion

The chalazion itself or its excision can damage the adjacent meibomian gland, and changes in the morphology of the meibomian gland can cause tear film instability and higher-order abnormalities [4, 28]. Large chalazion masses in the upper eyelid of children can lead to secondary ptosis and aggravated astigmatism [29], eventually increasing the risk of amblyopia. Donaldson et al. reported a case of occlusion amblyopia and secondary exotropia due to a chalazion mass in the upper eyelid in a 13-month-old infant [30]. Currently, there are no uniform guidelines on the choice and timing of treatment for a chalazion and conservative treatment has an effective rate of only 25–50% [31]. We investigated the refractive status of children with a chalazion aged 0.5–6 years and examined the effect of the site of origin, size, and the number of chalazion masses on the incidence，type， mean value and vector analysis of astigmatism to provide a reference for the selection and timing of treatment modalities for a chalazion. In this study, chalazia masses were commonly found in the middle-upper eyelid. Chalazia may lead to astigmatism, especially AR and OBL. According to different groups, through the comparison of the incidence of astigmatism, we found that in the middle - upper eyelid and the medial-upper eyelid in single chalazion was higher than that in the control group. The incidence of astigmatism increased significantly when the size of astigmatism was ≥3 mm. When the number of masses was 2, the incidence of astigmatism was the highest. By comparison of arithmetic mean and vector mean, we found that the medial-upper eyelid, middle-upper eyelid and medial-lower eyelid could bring more severe astigmatism, The 3-5 mm and >5 mm group will increase the severity of astigmatism, but the risk of astigmatism was higher when the size of the mass > 5 mm.

Descriptive statistics showed that a chalazion was more commonly in the upper eyelid than in the lower eyelid and most in the middle-upper eyelid, which was consistent with the findings of Bagheri et al. [25]. We hypothesized that this is related to the anatomy of the meibomian gland. Greiner [32] showed that the upper eyelid has more meibomian gland ducts (approximately 31 versus 26 in the upper and lower eyelid, respectively), more vesicles, and two times the secretory capacity than that of the lower eyelid. Further, the upper eyelid is elongated, while the lower eyelid is thicker and shorter (5.5 mm versus 2 mm). The development of a chalazion is associated with oversecretion or blocked drainage of the meibomian gland. Hence, a chalazion is more likely to form and worsen under conditions like meibomian gland dysfunction and blepharitis [4]. Between the chalazion and the group, the incidence rate of astigmatism was higher in the chalazion group (42.79%) than in the control group. Studies have confirmed that a chalazion can cause corneal astigmatism [15–18]. To the best of our knowledge, no studies have examined the incidence rate of chalazion-induced astigmatism to date. In addition, Moreover, the incidence rates of AR and OBL (20.83, 18.71%; 11.11, 5.76%, respectively) were significantly higher than those of the control group (13.25, 2.41%), suggesting that a chalazion is more likely to cause AR and OBL. This result is consistent with the findings of Ki et al. [17]. Mimouni reported that AR and OBL have a greater effect on the loss of UDVA in patients with emmetropia undergoing laser refractive surgery [33]. Yen-Shou Chou [34] revealed that AR and OBL are more likely to cause amblyopia.

According to different sites of groups, by the analysis of incidence, type, mean and vector of astigmatism, suggesting that the location of chalazion affects the occurrence of astigmatism, and the middle and inner side of upper eyelid are high-risk factors. Some studies analyzed the changes in corneal topography before and after chalazion removal and concluded that a chalazion increases irregularities in the corneal surface, increasing higher-order abnormalities and thus causing corneal astigmatism [16, 25, 28]. Previous studies have confirmed that a chalazion in the middle-upper eyelid can cause corneal astigmatism [15–18]; however, the effect of chalazion masses at other sites on astigmatism has not been explored. In the present study, we found that chalazion masses at all sites and in the middle-upper eyelid can also lead to astigmatism. We hypothesized that this is related to mass compression that alters corneal curvature and scleral tension [35].

According to different sizes of groups, the incidence of astigmatism in large and medium groups was higher than that in control group. The arithmetic mean and vector of large and medium astigmatism were higher than those of control group, and the difference between large and medium astigmatism was statistically significant. These results suggested that the size of chalazion masses affects the occurrence of astigmatism, and the influence of > 5 mm group is greater than that of 3-5 mm group, which was consistent with the findings of Park et al. [15]. Ki et al. systematically revealed the mechanical effect of a chalazion on corneal astigmatism and found that large chalazion masses in the upper eyelid compress the cornea, causing corneal astigmatism [17].

According to different numbers of groups, the incidence of astigmatism, arithmetic mean and vector of each group were higher than those of the control group. The incidence rate of astigmatism was significantly higher in the multiple chalazion group (49.31%) than in the single chalazion group (40.29%). In addition, the highest incidence rate of astigmatism was 56% with two masses; however, the incidence rate of astigmatism decreased with ≥3 masses, suggesting that the number of chalazion masses increases the risk of astigmatism. Moreover, the effect of a chalazion on astigmatism decreased with ≥3 chalazion masses. We hypothesized that this might be related to the dispersion of the mechanical effect of chalazion pressure exerted on the eyes. A study reported [36] that the tensile strength of the cornea was 3.81 ± 0.40 MPa, and the stress and strain values of the cornea were α = 42.81 ± 11.67 and β = 2.97 ± 0.21, respectively. Hence, the pressure imposed by a chalazion above these levels can cause corneal astigmatism.

This study has some limitations. First, we only investigated the effect of the site, size, and the number of chalazion masses in astigmatism without further exploring the specific mechanisms of astigmatism progression through corneal topography. The corneal topography of children aged > 3 years could be refined to understand the changes in corneal morphology in children with chalazion masses. Second, only the preoperative refractive status of children with chalazion masses were available, but there was no postoperative refractive data of the children, which will be further supplemented in future studies. In future studies, we will refine preoperative and postoperative corneal topography and diopter examinations for children over 3 years old to investigate the specific mechanisms of chalazia-induced astigmatism.

## Conclusion

Children are more likely to develop chalazion masses in the middle-upper eyelid. A chalazion can also lead to astigmatism, especially AR and OBL. A chalazion in the middle-upper eyelid is more likely to cause astigmatism than those in other sites. Moreover, astigmatism is related to the site of the origin, size, and number of chalazion masses. A chalazion in the middle-upper eyelid, chalazion masses ≥3 mm, and multiple chalazion masses (especially two chalazion masses) are risk factors of astigmatism. Prompt invasive treatment is recommended if conservative treatment is ineffective to avoid further harm to the visual acuity due to chalazion-induced astigmatism in children.

## Supplementary Information

**Additional file 1.**

## Data Availability

All data generated or analyzed during this study are included in this published article and its supplementary information files.

## Abbreviations

WR: With-the-rule astigmatism
AR: Against-the-rule astigmatism
OBL: Oblique astigmatism; non-WR = AR + OBL
UDVA: Uncorrected distance visual acuity
AAPOS: American Academy of Pediatrics Committee on Ophthalmology and Strabismus

## References

[1] Unal M (2008). Chalazion treatment. Orbit..

[2] Liang L, Ding X, Tseng SC (2014). High prevalence of demodex brevis infestation in chalazia. Am J Ophthalmol.

[3] Evans J, Vo KBH, Schmitt M (2021). Chalazion: racial risk factors for formation, recurrence, and surgical intervention. Can J Ophthalmol.

[4] Yin Y, Gong L (2017). The evaluation of meibomian gland function, morphology and related medical history in Asian adult blepharokeratoconjunctivitis patients. Acta Ophthalmol.

[5] Woo YR, Cho M, Ju HJ, Bae JM, Cho SH, Lee JD (2021). Ocular comorbidities in Rosacea: a case-control study based on seven institutions. J Clin Med.

[6] Chen L, Chen X, Xiang Q, Zheng Y, Pi L, Liu Q (2014). Prevalence of low serum vitamin a levels in young children with chalazia in Southwest China. Am J Ophthalmol.

[7] Ilhan C (2021). Retrospective investigation of peripheric blood sampling in pediatric chalazion patients. Int Ophthalmol.

[8] Donmez O, Akova YA (2021). Pediatric ocular acne Rosacea: clinical features and long term follow-up of sixteen cases. Ocul Immunol Inflamm.

[9] Wu AY, Gervasio KA, Gergoudis KN, Wei C, Oestreicher JH, Harvey JT (2018). Conservative therapy for chalazia: is it really effective?. Acta Ophthalmol.

[10] Singhania R, Sharma N, Vashisht S, Dewan T (2018). Intralesional triamcinolone Acetonide (TA) versus incision and curettage (I & C) for medium and large size Chalazia. Nepal J Ophthalmol.

[11] Paik JS, Kim SA, Park SH, Yang SW (2016). Refractive error characteristics in patients with congenital blepharoptosis before and after ptosis repair surgery. BMC Ophthalmol.

[12] Herlihy EP, Kelly JP, Sidbury R, Perkins JA, Weiss AH (2016). Visual acuity and astigmatism in periocular infantile hemangiomas treated with oral beta-blocker versus intralesional corticosteroid injection. J AAPOS..

[13] AlHarkan DH (2020). Management of vernal keratoconjunctivitis in children in Saudi Arabia. Oman J Ophthalmol.

[14] Ahuja P, Dadachanji Z, Shetty R, Nagarajan SA, Khamar P, Sethu S, D'Souza S (2020). Relevance of IgE, allergy and eye rubbing in the pathogenesis and management of Keratoconus. Indian J Ophthalmol.

[15] Park YM, Lee JS (2014). The effects of chalazion excision on corneal surface aberrations. Cont Lens Anterior Eye.

[16] Sabermoghaddam AA, Zarei-Ghanavati S, Abrishami M (2013). Effects of chalazion excision on ocular aberrations. Cornea..

[17] Jin KW, Shin YJ, Hyon JY (2017). Effects of chalazia on corneal astigmatism: large-sized chalazia in middle upper eyelids compress the cornea and induce the corneal astigmatism. BMC Ophthalmol.

[18] Ilhan C, Ozgul Yilmazoglu M, Yilmazbas P (2019). The effects of chalazion surgery on intraocular pressure and corneal topography. Int Ophthalmol.

[19] Samaras K, Lindfield D, Saleh GM, Poole TR (2008). Chalazion-induced hypermetropia: a topographic illustration. Eye (Lond).

[20] Cosar CB, Rapuano CJ, Cohen EJ, Laibson PR (2001). Chalazion as a cause of decreased vision after LASIK. Cornea..

[21] Woltsche N, Werkl P, Posch-Pertl L, Ardjomand N, Frings A (2019). Astigmatismus [Astigmatism]. Ophthalmologe..

[22] Eydelman MB, Drum B, Holladay J, Hilmantel G, Kezirian G, Durrie D, Stulting RD, Sanders D, Wong B (2006). Standardized analyses of correction of astigmatism by laser systems that reshape the cornea. J Refract Surg.

[23] Misra N, Khanna RC, Mettla AL, Marmamula S, Keeffe JE (2021). Agreement and diagnostic accuracy of vision screening in preschool children between vision technicians and spot vision screener. Indian J Ophthalmol.

[24] Kara C, Petriçli İS (2020). Comparison of photoscreening and autorefractive screening for the detection of amblyopia risk factors in children under 3 years of age. J AAPOS.

[25] Bagheri A, Hasani HR, Karimian F, Abrishami M, Yazdani S (2009). Effect of chalazion excision on refractive error and corneal topography. Eur J Ophthalmol.

[26] Kunert KS, Russmann C, Blum M, Sluyterman VLG (2013). Vector analysis of myopic astigmatism corrected by femtosecond refractive lenticule extraction. J Cataract Refract Surg.

[27] Holladay JT, Dudeja DR, Koch DD (1998). Evaluating and reporting astigmatism for individual and aggregate data. J Cataract Refract Surg.

[28] Fukuoka S, Arita R, Shirakawa R, Morishige N (2017). Changes in meibomian gland morphology and ocular higher-order aberrations in eyes with chalazion. Clin Ophthalmol.

[29] Wang Y, Xu Y, Liu X, Lou L, Ye J (2018). Amblyopia, strabismus and refractive errors in congenital ptosis: a systematic review and meta-analysis. Sci Rep.

[30] Donaldson MJ, Gole GA (2005). Amblyopia due to inflamed chalazion in a 13-month old infant. Clin Exp Ophthalmol.

[31] Goawalla A, Lee V (2007). A prospective randomized treatment study comparing three treatment options for chalazia: triamcinolone acetonide injections, incision and curettage and treatment with hot compresses. Clin Exp Ophthalmol.

[32] Greiner JV, Glonek T, Korb DR, Whalen AC, Hebert E, Hearn SL (1998). Volume of the human and rabbit meibomian gland system. Adv Exp Med Biol.

[33] Mimouni M, Nemet A, Pokroy R, Sela T, Munzer G, Kaiserman I (2017). The effect of astigmatism axis on visual acuity. Eur J Ophthalmol.

[34] Chou YS, Tai MC, Chen PL, Lu DW, Chien KH (2014). Impact of cylinder axis on the treatment for astigmatic amblyopia. Am J Ophthalmol.

[35] Torres-Netto EA, Kling S (2022). Corneal strain induced by Intracorneal ring segment implantation visualized with optical coherence Elastography. J Refract Surg.

[36] Zeng Y, Yang J, Huang K, Lee Z, Lee X (2001). A comparison of biomechanical properties between human and porcine cornea. J Biomech.

